# A contrasting function for miR-137 in embryonic mammogenesis and adult breast carcinogenesis

**DOI:** 10.18632/oncotarget.4218

**Published:** 2015-05-20

**Authors:** Jong-Min Lee, Kyoung-Won Cho, Eun-Jung Kim, Qinghuang Tang, Kye-Seong Kim, Cheryll Tickle, Han-Sung Jung

**Affiliations:** ^1^ Division in Anatomy and Developmental Biology, Department of Oral Biology, Oral Science Research Center, BK21 PLUS Project, Yonsei University College of Dentistry, Seoul, Korea; ^2^ Department of Radiology, Seoul National University Hospital, Seoul, Korea; ^3^ Graduate School of Biomedical Science and Engineering, College of Medicine, Hanyang University, Seoul, Korea; ^4^ Department of Biology and Biochemistry, University of Bath, Bath, UK; ^5^ Oral Biosciences, Faculty of Dentistry, The University of Hong Kong, Hong Kong SAR

**Keywords:** mammary gland development, breast cancer, miR-137, tumour suppressor, tachykinin-1

## Abstract

MicroRNAs are differentially expressed in breast cancer cells and have been implicated in cancer formation, tumour invasion and metastasis. We investigated the miRNA expression profiles in the developing mammary gland. MiR-137 was expressed prominently in the developing mammary gland. When the miR-137 was over-expressed in the embryo, the mammary epithelium became thickened. Moreover, genes associated with mammary gland formation such as *Tbx3* and *Lef1* were not expressed. This suggests that miR-137 induces gland formation and invasion. When miR-137 was over-expressed in MDA-MB-231 cells, their ability to form tumours in adult mice was significantly reduced. These data support miR-137 decides epithelial cell behavior in the human breast cancer. It also suggests that miR-137 is a potential therapeutic target for amelioration of breast cancer progression.

## INTRODUCTION

Mammary gland development in mouse embryos is used as a model for breast cancer and can be divided into a series of stages [1-3]. In the initiation stage (in E10.5 mouse embryos), bilateral mammary lines are formed and epidermal cells of the mammary line become columnar and multilayered, defining the mammary ridge. At E11.5, five pairs of mammary placodes form at specific locations along the mammary line and these placodes then begin to invade the underlying mesenchyme (E12.5) to form a spherical epithelial bud enveloped by dense mammary mesenchyme and connected to the epidermis by a short stalk (E13.5) [4]. Subsequently, the epithelial bud grows and undergoes branching morphogenesis in female mice. Many genes expressed in the developing mouse mammary gland are also expressed in adult human breast cancer, including genes encoding molecules involved in signaling e.g. the estrogen receptor is expressed in the mesenchyme surrounding the invasive mammary epithelial bud [5] and various transcription factors [6-10]. For example, *Tac1*, the gene encoding Tachykinin-1, the precursor protein for neuroendocrine peptides, is expressed in the mammary mesenchyme during embryonic development [11-13] and the level of *Tac1* expression in breast cancers is directly proportional to their aggressiveness [14, 15]. Another example is the GATA family zinc-finger transcription factor, GATA3, a crucial regulator of luminal differentiation during mammary gland development [16, 17] which is expressed within invasive breast carcinomas [18].

Alterations in several genes, including those encoding microRNAs (miRNAs), accumulate in cancer by a complex and multistep process [19]. MiRNAs are 19- to 25-nucleotide single-stranded RNA molecules that function as post-transcriptional regulators of target genes expression [20, 21]. Recent studies have reported that miRNAs may act as either tumour suppressors or oncogenes [22,23] and there is interest in identifying miRNAs associated with breast cancer as they could be novel therapeutic targets. Here we investigated which miRNAs are expressed in the early development of mouse mammary glands by comparing the 3^rd^ mammary gland and the inter mammary gland region from E13.5 mouse embryos. We found that miRNA-137 (miR-137) is highly expressed in the 3^rd^ mammary gland at E13.5 and over-expressing it in the flank region of developing mouse embryos perturbed invasion of the epithelial mammary bud.

Down-regulated miR-137 has been observed in various cancers such as colorectal cancer, gastric cancer, oral cancer, and squamous cell carcinoma of the head and neck [24-27]. A recent study also reported that miR-137 impairs proliferation and migration of cells of a breast cancer cell line by targeting expression of the nuclear receptor estrogen-related receptor alpha (ERRα) [28], suggesting that miR-137 may suppress the formation of breast cancer. To confirm this possibility *in vivo*, we over-expressed miR-137 in cells of a breast cancer cell line, then inoculated them into nude mice and found that their ability to form tumours was reduced.

The outcomes of this study reveal that miR-137 not only perturbs embryonic mammary gland development but also inhibits tumour formation by human breast cancer cells. These results bolster the idea that the embryonic mouse mammary gland is a good model for human breast cancer.

## RESULTS

### Microarray profiling of embryonic mouse mammary glands

**Figure 1:**
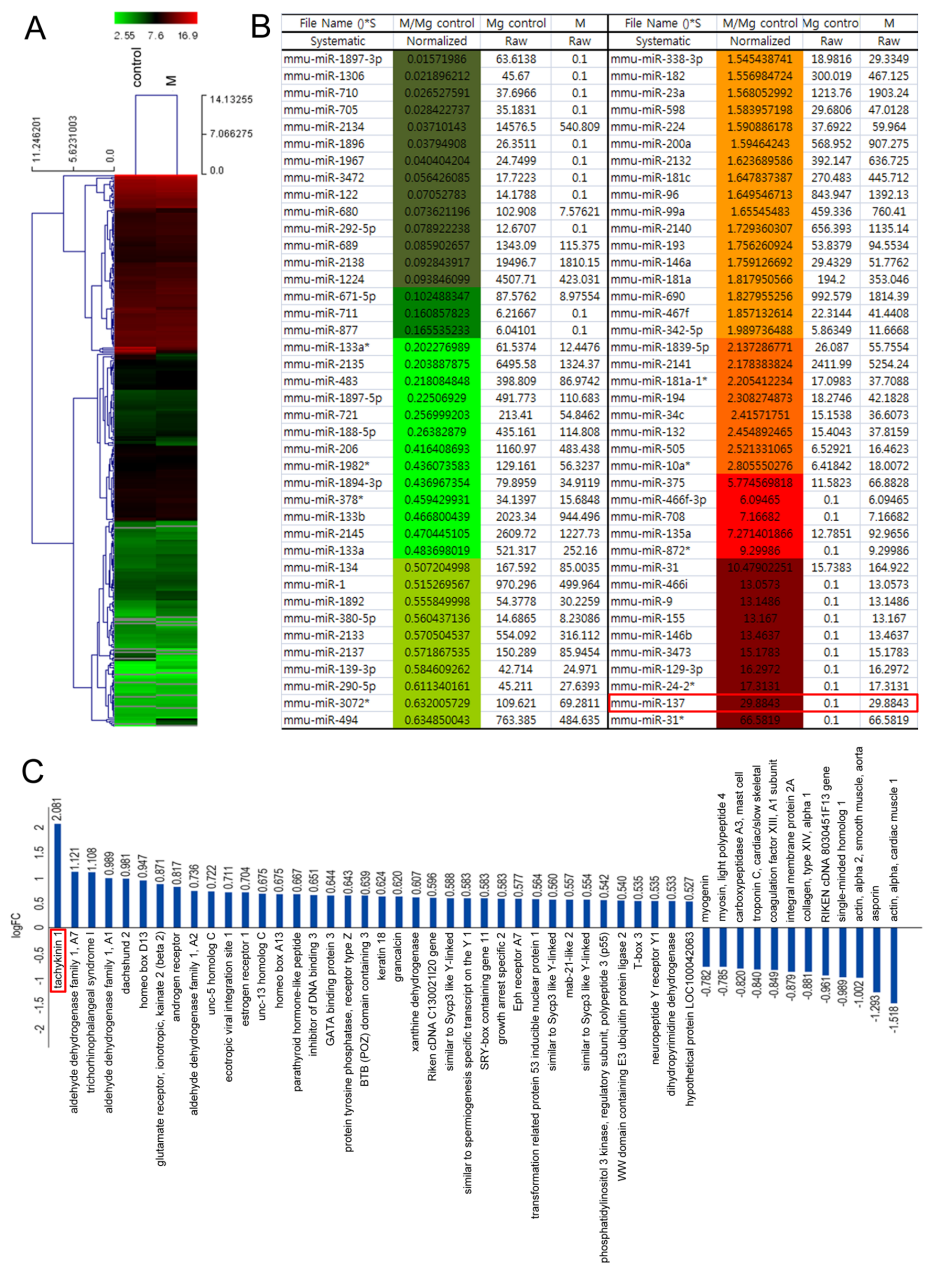
Heat-map of 3^rd^ mammary gland and inter mammary gland region miRNA expression illustrating cluster-analysis of the expression data **A., B.** The signal intensity converted to log2 is reflected in the color scale such that dark green, green and red show low, medium and strong miRNA expression, respectively. The dendrogram on the left shows hierarchical similarity. **C.** Microarray data sorting for protein-coding genes related to mammary gland development.

To identify miRNAs expressed in the embryonic mouse mammary gland during invagination of the epithelial bud, we used miRNA microarrays to compare the 3^rd^ mammary gland and inter mammary gland flank region dissected from E13.5 mouse embryos (Supplementary Figure S1A-S1C). This analysis showed that expression of many miRNAs is correlated to mammary gland development (Figure 1A and 1B). Among these, the expression level of miR-137 was increased about 30 fold in the 3^rd^ mammary gland compared to inter mammary gland region (red box in Figure 1B). This high level of expression in the 3^rd^ mammary gland forming region was confirmed by carrying out *in situ* hybridization using a miR-137 locked nucleic acid (LNA) probe to detect miR-137 transcripts (Supplementary Figure S1D). We also carried out further microarray analysis using tissue dissected from mouse embryos in the same way to screen for protein-coding genes differentially expressed in the developing gland. As expected, we found many genes already known to be highly expressed in developing mouse gland at similar stages [5]. For example, *ER1* and *Gata3* were highly expressed in 3^rd^ mammary gland compared to the intermammary gland region (Figure 1C). The gene that we found to be most highly expressed in the 3^rd^ mammary gland at E13.5 was *Tac1* (red boxes in Figure 1C).

### Relationship between miR-137 and mammary gland development

**Figure 2:**
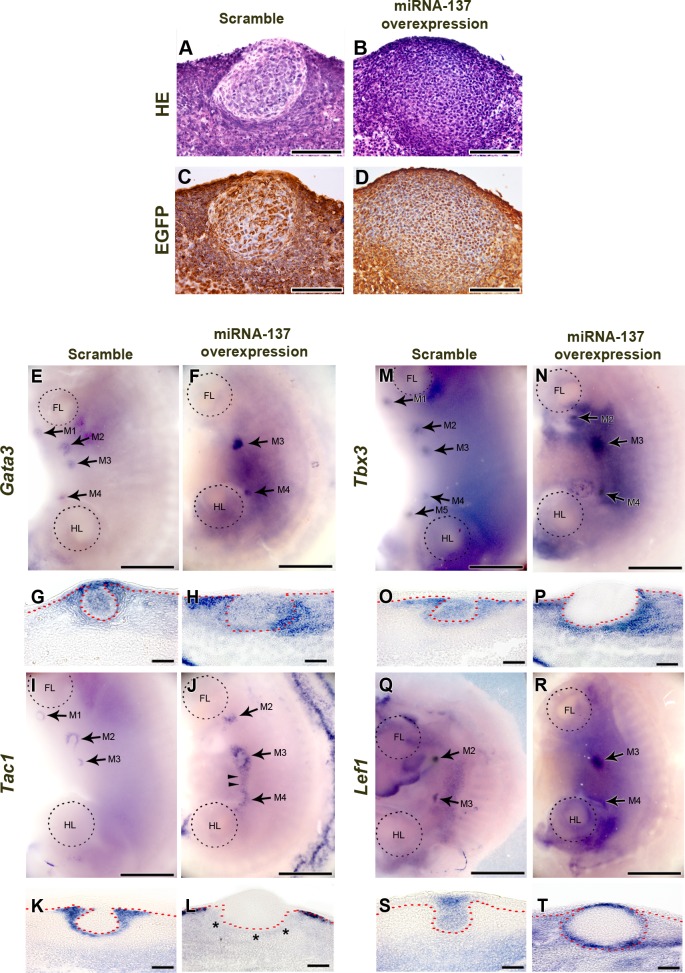
Function of miR-137 during mammary gland development **A., B.** HE staining of sections of mammary glands that developed after miR-137 over-expression, shows that normal invasion has not taken place although the placode has thickened. **C.**, **D.** EGFP IHC indicates that scrambled miRNA and miR-137 have been successfully over-expressed. **E.**-**H.** *Gata3* expression is disrupted by over-expressed miR-137. **I.**-**L.** MiR-137 over-expression alters *Tac1* expression. (M-T) Expression patterns of the mammary gland markers, *Tbx3* and *Lef1*, are changed after miR-137 treatment. Arrows, mammary bud. Arrowheads, ectopic *Tac1* expression. Asterisk, disrupted *Tac1* expression region ; FL, fore limb; HL, hind limb; Scale bar, A-D, G, H, K, L, O, P, S, T, 100 μm; E, F, I, J, M, N, Q, R, 1 mm.

To investigate the function of miR-137 in mouse mammary gland development, we over-expressed miR-137 using a lentiviral system in the flank region of E11.0 mouse embryos and then cultured the flank for 72 h. As controls, we over-expressed scrambled miRNA. The lentiviral system also incorporated an EGFP cassette and successful over-expression of miR-137 or the control scrambled miRNA was confirmed by immunohistochemistry (IHC) using EGFP antibody and real-time quantitative polymerase chain reaction (RT-qPCR) (Figure 2C and 2D; Supplementary Figure S2A). In the controls, well-developed mammary buds were observed. With the over-expression of miR-137, thickened epithelium was observed in mammary placode region. However, this enlarged epithelium showed different cell morphology from normal mammary placode cells and they did not protrude into the underlying mesenchyme to give a spherical bud (compare Figure 2A and 2C with Figure 2B and 2D; see also for example Figure 2O versus 2P). To determine whether miRNA-137 inhibits or simulates expression of genes involved in mammary gland formation, we applied miR-137 to cultured mouse flanks and examined expression of *Gata3*, T-box transcription factor *Tbx3*, *Tac1*, and *Lef1* (lymphoid enhancing factor 1) (Figure 2E-2T).

**Figure 3:**
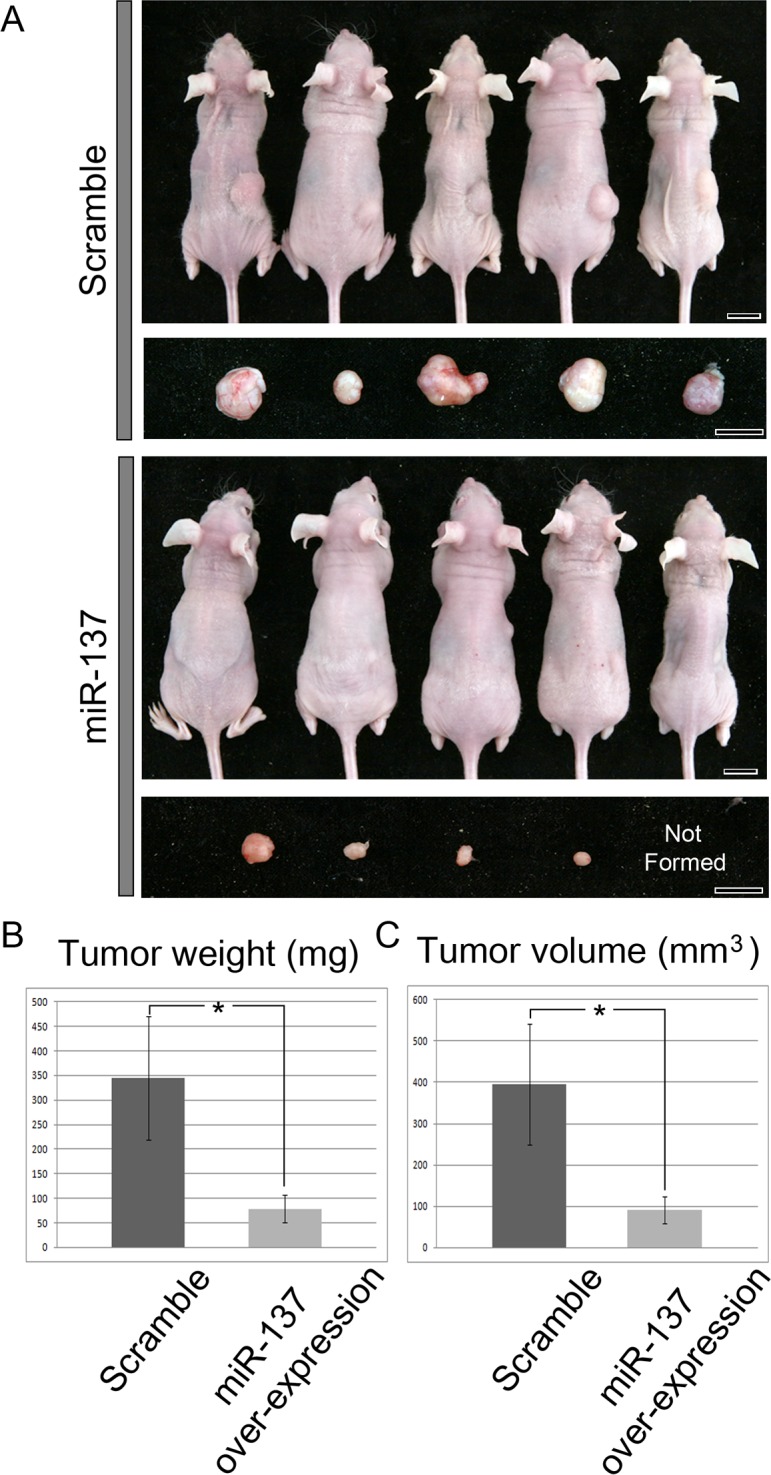
Tumour formation after SC inoculation of MDA-MB-231 cells over-expressing scrambled miRNA (controls) or over-expressing miR-137 **A.** Cells over-expressing miR-137 form smaller tumours than cells over-expressing scrambled miRNA. **B.** Tumour weight is markedly reduced after miR-137 over-expression **C.** as is tumour volume compared to controls. Scale bar, 1 cm.

Surface views of the embryos showed that the *Gata3* expression pattern was ring shaped, in the mammary glands at E12.5. However, at E13.5, expression of *Gata3* was seen in entire mammary gland region (compare Figure 2E and 2G with Supplementary Figure S3S and V). *Gata3* expression pattern was altered by miR-137 over-expression. Mesenchymal expression of *Gata3* was induced but epithelial expression was reduced by miR-137 over-expression (Figure 2F and 2H). *Gata3* was expressed in the center of mammary epithelial, surface ectoderm and mammary mesenchyme just beneath epithelium of mammary bud in control (Figure 2G). However, *Gata3* expression was lost its typical pattern in mammary epithelium and surface ectoderm after miR-137 over-expression (Figure 3H). *Gata3* was observed in the flank mesenchyme besides the mammary bud (Figure 2F) extending both ventrally and dorsally after miR-137 over-expression (Figure 2H).

*Tac1* is also known to be expressed only in mammary mesenchyme around in the developing mammary bud (Supplementary Figure S3G-S3L; ref 11-13) and this expression pattern was not changed by over-expression of scrambled miRNA (compare Figure 2I, 2K and Supplementary Figure S3G, S3J). Ectopic expression of *Tac1* was detected between 3^rd^ and 4^th^ mammary gland after miR-137 over-expression (Figure 2I and 2J arrowheads). In transverse section showed that *Tac1* was expressed in mesenchyme under surface ectoderm region but it was not expressed mesenchyme below mammary bud after miR-137 over-expression (Figure 2K and 2L asterisks).

Wnt signaling correlates expression of the transcription factor, *Tbx3*, which is essential for epithelial-mesenchymal interaction with *Lef1* during early mammary gland development [30, 31]. In control, *Tbx3* was expressed in mammary bud, mammary gland mesenchyme and dorsal part of flank in mouse embryo (Figure 2M and 2O). After miR-137 over-expression, *Tbx3* expression pattern was similar as control in surface view of the dorsal part embryo flank (Figure 2N). However, transverse section showed that *Tbx3* was completely obliterated in the mammary epithelium (Figure 2P). In addition, *Tbx3* expression was induced in mammary mesenchyme and dermal mesenchyme region in dorsal part of flank (Figure 2P).

In the controls, expression of the *Lef1* was observed in mammary epithelium from initiation (E10) to bud stage (E13) [31]. It was clearly observed in mammary epithelial buds in control (Figure 2Q and 2S). However, *Lef1* expression was detected in broad region of mammary gland than control after miR-137 over-expression (Figure 2R). Moreover, *Lef1* was not expressed in mammary epithelium except surface ectoderm and the margin of the enlarged mammary placodes between mammary epithelium and mesenchyme (Figure 2T). In addition, ectopic *Lef1* expression was observed in mesenchyme around mammary gland after miR-137 over-exrpression (Figure 2T).

All these results indicate that over-expression of miRNA-137 suppresses development of the mammary gland in mouse embryos.

### *In vivo* tumour formation assay after miR-137 over-expression

Recent work has shown that miR-137 impairs proliferation and migration of MDA-MB-231 human breast cancer cells in culture [28]. In order to investigate the relationship between miR-137 and breast tumour growth *in vivo*, MDA-MB-231 cells over-expressing miR-137 were inoculated subcutaneously (SC) in nude mice (*n* = 34) and formation of tumours were monitored 7 weeks later. Over-expression of miR-137 in the cells was confirmed by RT-qPCR (Supplementary Figure S2B). As a control, MDA-MB-231 cells over-expressing scrambled miRNA were inoculated (*n* = 16). The tumours formed by miR-137 over-expressing cells were noticeably smaller than those formed by cells transfected with the scrambled miRNA (Figure 3A). In fact, in 3 of the mice injected with MDA-MB-231 cells over-expressing miR-137 no tumours formed at all. In order to quantitate these results, we measured tumour weight and volume. Both weight and volume of tumours developing from miR-137 over-expressing cells were significantly reduced compared with tumours developing from cells over-expressing scrambled miRNA (Figure 3B and 3C). Thus, these results showed that miR-137 can suppress tumour formation *in vivo*.

### Expression pattern of angiogenesis and tumour suppressor markers after miR-137 overexpression

To explore the mechanisms involved in suppressing tumour formation when miR-137 is over-expressed in MDA-MB-231 cells, we performed IHC to monitor cell proliferation, angiogenesis and expression of tumour suppressors. Cell proliferation was reduced in the tumours formed by miR-137 over-expressing cells. A large number of Ki67-positive proliferating cells were observed in tumours formed by cells expressing the scrambled miRNA compared to tumours formed by cells over-expressing miR-137 (Supplementary Figure S4A-S4D). The number of Ki67-positive cells was counted in scramble and miR-137 over-expression group. The numbers of Ki67-positive cells were 38.57 cells/100 × 100 μm and 14.14 cells/100 × 100 μm in scramble miRNA and miR-137 over-expressing group, respectively (Supplementary Figure S4E).

**Figure 4:**
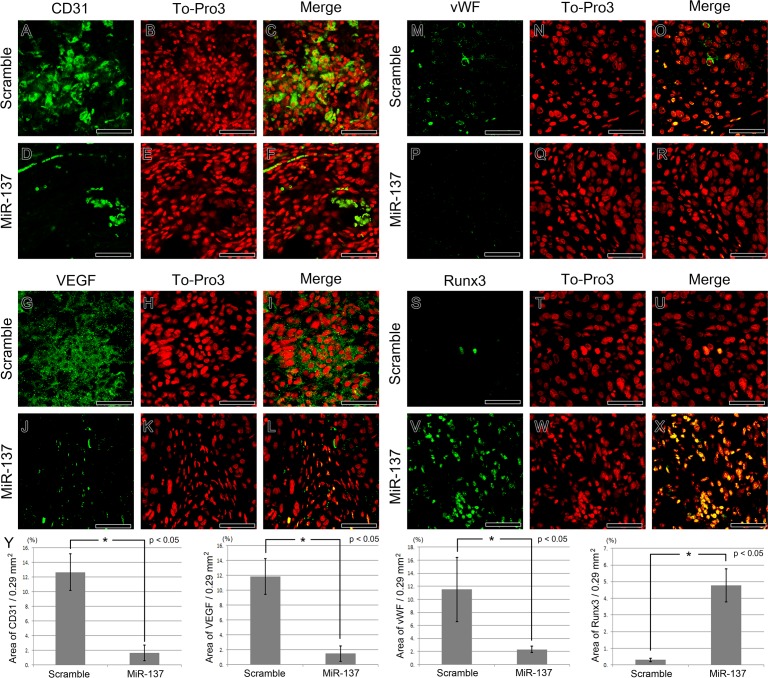
IHC of vasculogenesis and tumour suppressor markers after miR-137 over-expression **A.**-**F.** Sinusoidal endothelial cell marker CD31 is reduced after miR-137 over-expression. **G.**-**L.** Over-expressed miR-137 inhibits VEGF expression. **M.**-**R.** vWF expression is decreased in breast cancer after over-expression of miR-137 in MDA-MB-231. **S.**-**X.** Tumour suppressor Runx3 expression is substantially increased after miR-137 treatment. **Y.** The area of CD31, VEGF, vWF and Runx3 expression in the scramble miRNA and miR-137 over-expression group (*n* = 7 for each). IHC staining positive areas Scale bar, 100 μm.

**Figure 5:**
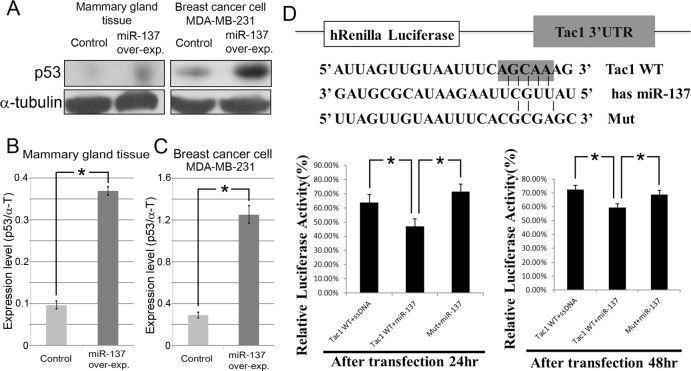
MiR-137 induces p53 expression in mammary gland tissue and breast cancer **A.**-**C.** Expression level of p53 is increased in mammary gland tissue and MDA-MB-231 cells after over-expressing miR-137. **D.** A luciferase assay result indicating that *Tac1* is a direct target of miR-137.

In addition, we found that angiogenesis was markedly reduced after miR-137 over-expression. Staining for the sinusoidal endothelial cell marker CD31 indicated that there were a smaller number of blood vessels in the tumours made by miR-137 over-expressing MDA-MB-231 cells (Figure 4A-4F) and furthermore the expression of vasculogenesis markers, VEGF and vWF were dramatically reduced (Figure 4G-4L; Figure 4M-4R). On the other hand, expression of known tumour suppressors, Runx3 and p53, was increased in the tumours formed by miR-137 over-expressing cells (Figure 4S-4X; Figure 5A-5C). The area of CD31, VEGF, vWF and Runx3 expression in the scramble miRNA and miR-137 over-expression group was measured (*n* = 7 for each). The CD31, VEGF and vWF positive area was reduced in the miR-137 over-expression group than in those treated with scramble miRNA treated group. Runx3 expression region was increased in miR-137 treated group compared to control group (Figure 4Y).

Moreover, the cells in the tumours formed from miR-137 over-expressing cells induced the epithelial marker E-cadherin expression (Supplementary Figure S5A-S5F) but mesenchymal marker, Vimentin expression was reduced compare to control (Supplementary Figure S5G-S5L).

MiR-137 binding to *Tac1* was examined by measuring luciferase activity with a dual-luciferase assay vector containing cloned 3′UTR of *Tac1* transcripts (Figure 5D). Wild-type (WT) *Tac1* reporters showed an inhibition of Renilla luciferase activity compared with the mutant reporter (Mut), with mismatched sequences inserted into the seed sequences of the predicted miR-137 binding site (Figure 5D). Luciferase assay results indicate that *Tac1*, which confers a poor prognosis in breast cancer [14, 15], was the directly correlated to miR-137 (Figure 5D).

## DISCUSSION

From screens of tissue from mouse embryos for miRNAs differentially expressed in the developing mammary gland, we identified miR-137 was highly expressed. We also identified, via screening of the same tissues, that many protein-coding genes were already known to be expressed in the developing mammary gland and in breast tumours. Upon over-expression of miR-137 in mouse embryo flank during organ culture, the mammary epithelium thickened but it did not express several key genes known to be required for gland development and it also failed to invaginate into the underlying mesenchyme. MiR-137 is also highly expressed in an epithelial cell line derived from the adult human breast. When we over-expressed miR-137 in this breast cancer cell line, tumour formation *in vivo* was suppressed.

We identified miR-137 as a miRNA highly expressed in the developing mouse mammary gland at a stage when the mammary placode has invaginated the underlying mesenchyme to form a spherical bud. Our data and that of others [5] reveal that around this stage in development, many genes encoding proteins known to be associated with breast cancer are expressed in the gland. Thus, the molecular analysis of the mouse embryonic mammary gland is a good strategy for identifying genes that are potentially involved in breast cancer and any miRNAs identified from the mammary gland are good candidates for miRNAs associated with breast cancer.

*ER1*, *GATA3* and *Trps1* are among the genes that are highly expressed in the E13.5 mouse mammary gland and have also been implicated in human breast cancer [5, 16-18]. These genes were also identified by microarray analysis of E12.5 mammary glands. In addition, our analysis identified genes encoding proteins involved in retinoic acid signaling which appears to be involved in gland initiation [32]. Some genes identified are expressed in the mesenchyme and some in the epithelial bud and it is well-established that there are complex reciprocal signaling correlations between these tissues during normal development.

Our functional analysis suggests that miR-137 is involved in modulating early stages of mammary gland formation in the embryo especially invasion. When miR-137 is over-expressed, mammary placode formed and thickened but failed to invade the underlying mesenchyme and did not express either *Tbx3* or *Lef1* in mammary epithelium. *Tbx3* mutations are found in Ulnar mammary syndrome in human patients, which is characterized by mammary gland hypoplasia [33, 34]. In *Tbx3* knockout (KO) mouse embryos, mammary placode do not form and expression of *Lef1* is absent [34]. *Lef1* expression is one of the earliest molecular markers of placode formation and is also maintained in the epithelial cells during invasion of the epithelial bud. Thus, the failure of the mammary placode invagination by loss of *Tbx3* and *Lef1* after miR-137 over-expression suggests that miR-137 correlated the early stages of mammary gland development. *Tac1* and *Gata3* genes whose expression is also altered by over-expression of miR-137 are also detected at early bud stages. Moreover, luciferase assay results indicate that *Tac1*, which confers a poor prognosis in breast cancer [14, 15], was the direct target of miR-137. These data suggest that miR-137 is a potential therapeutic target for breast cancer by controlling of *Tac1* expression.

In breast cancer cells, miR-137 has been shown to target expression of ERRα [28]. It is not clear whether ERRα could be the target of miR-137 in the developing mouse mammary gland. Interestingly, *ER1* is expressed in the mesenchyme surrounding the mammary bud at E12.5 [5] and is a target of miR-206, which is also expressed in the mesenchyme at E11.5 and E12.5 (earlier than miR-137) but not at E13.5 [35]. When miR-206 was over-expressed in the same flank culture system used here, mammary placode development was very severely retarded; the placode formed but did not thicken [35]. This is consistent with miR-206 having a function at a slightly earlier stage in mammary gland development than miR-137.

Our results also showed that over-expression of miR-137 reduced tumour formation *in vivo*. Interestingly, we observed that cell proliferation of subcutaneously inoculated breast cancer cells was reduced when miR-137 was over-expressed. Moreover, epithelial-mesenchymal transition (EMT) was inhibited in the tumours formed from miR-137 over-expressing MDA-MB-231 cells. These results indicate that miR-137 have multiple tumour suppress function including prevention of the cell proliferation, epithelial invasion and EMT.

Our focus on miR-137 initially arose from screening for genes differentially expressed in embryonic mammary glands and this shows how the study of the developing embryonic mammary gland can provide new perspectives on breast cancer.

## MATERIALS AND METHODS

All experiments were performed according to the guidelines of the Intramural Animal Use and Care Committee of Yonsei University College of Dentistry.

### Animals

Adult Institute of Cancer Research Research; Caesarian Derived-1 (ICR; CD-1) mice were housed in a temperature-controlled room (22°C) under artificial illumination (lights on from 0500 to 1700 hours) and 55% relative humidity. The mice had access to food and water ad libitum. Embryos were obtained from time-mated pregnant mice. E0 was designated as the day a vaginal plug was confirmed. Embryos at developmental stages E11.0, E12.5 and E13.5 were used in this study. To study tumour formation, subcutaneous (SC) inoculation of 2.0×10^6^ MDA-MB-231 cells was performed in the BALB/c nude mice (Orientbio, Korea). No immunosuppressive medication was used. Tumour size was measured seven weeks after SC inoculation. The host nude mice were sacrificed, and tumours were dissected for analysis.

### MiRNA microarray analysis

For mammary gland miRNAs, the synthesis of target miRNA probes and hybridization were performed using Agilent's miRNA Labelling Reagent and Hybridization kit (Agilent Technology, USA) according to the manufacturer's instructions. Briefly, 100 ng of total RNA from each region (the 3^rd^ mammary gland and the inter-mammary gland region) at E13.5 was dephosphorylated with ∼15 Units of calf intestine alkaline phosphatase (CIP), followed by RNA denaturation with ∼40% DMSO and a 10 min incubation at 100 °C. Dephosphorylated RNA was ligated with pCp-Cy3 mononucleotide and purified with MicroBioSpin 6 columns (Bio-Rad, USA). After purification, labelled samples were resuspended with Gene Expression blocking Reagent and Hi-RPM Hybridization buffer, followed by boiling for 5 min at 100 °C and 5 min chilled on ice. Finally, denatured labelled probes were pipetted onto the assembled Agilent miRNA Microarray (15K) and hybridized for 20 hours at 55 °C with rotation at 20 rpm in an Agilent Hybridization oven (Agilent Technology, USA). The hybridized microarrays were washed as the manufacturer's washing protocol (Agilent Technology, USA). Location of array raw data; GEO (http://www.ncbi.nlm.nih.gov/geo/query/acc.cgi?acc=GSE66546)

### Data acquisition and analysis

The hybridized images were scanned using Agilent's DNA microarray scanner and quantified with Feature Extraction Software (Agilent Technology, Palo Alto, CA). All data normalization and selection of fold-changed genes were performed using GeneSpringGX 7.3 (Agilent Technology, USA). The averages of normalized ratios were calculated by dividing the average of normalized signal channel intensity by the average of normalized control channel intensity. Functional annotation of genes was performed according to the Gene OntologyTM Consortium (http://www.geneontology.org/index.shtml) by GeneSpringGX 7.3.

### Lentivirus production by transfecting 293T cells

We used miExpress^TM^ precursor miRNA expression clone (GeneCopoeia^TM^, USA) for miR-137 over-expression in mouse embryo flank and MDA-MB-231 cell. This clone is pEZX-MR04 vector system which carries EGFP and CMV promoter. The pEZX-MR04 vector confers resistance to puromycin (5-15 μg/ml) in transduced or transfected cells. The minimum antibiotic concentration to use is the lowest concentration that kills 100% of the cells in 3-6 days from the start of antibiotic selection. Finally we used 10 μg/ml purimycin for transfection of lentivirus production. Lentiviral production was carried out by transfecting human embryonic kidney 293T cells with lentivital and packaging vectors. For viral tittering, 5 × 10^4^ 293T cells were seed into 24-well culture plate with DMEM (10% FBS, 1% pen-strep) and counted the EGFP expressing cells or colonies of cells. EGFP positive colonies X 125 (dilution factor) X 40 = 2.76 × 10^5^ TU/mL. For each 100 mm dish, helper plasmid pCD-NL/BH*ΔΔΔ 3 μg, envelope pLTR-G 300 ng, target miR-137 expressing lentivirus DNA 3 μg were mixed in 500 μl transfection reagent FuGENE HD (Roche). This mixture was incubated at room temperature for 20 min, and then added to 293T cells. The medium was changed after 4 h into culture medium containing 30% FBS. The resulting supernatant was collected 48 h, filtered through syringe driven filter Unit 0.45μg (Millipore, Cat No. SLHV 033 RS) and concentrated by Amicon Ultra-15 Centrifugal Filter Devices (Millipore, Cat No. UFC910024). Virus medium was used for *in vitro* organ and breast cancer cell culture.

### Over-expression of miR-137 in *in vitro* organ culture and breast cancer cells

During cell culture (MDA-MB-231), miR-137 expressing lentiviral vector was transduced into cells. Concentrated miR-137 expressing lentivirus was added 1% (v/v) in culture medium containing polybrene (Santa Cruz Biotechnology). ICR mouse embryos were isolated at E11.0 and placed in culture medium (BGJb; Sigma, USA). The culture medium was supplemented with 20 μg/mL (v/v) ascorbic acid (Sigma, USA) and 1% (v/v) penicillin/streptomycin. Individual embryos were dissected into left and right halves using fine tungsten needles to bisect the neural tube. The left flank was designated the experimental tissue, and the right acted as control. Each flank was placed onto filter membranes (Track-etch, 1.0 μm pore; Whatman Nuclepore) that were supported on stainless steel grids in sterile culture dishes. Tissues were cultured in the air–medium interface at 37 °C and 5% CO_2_ for 12 h and 48 h by using a slight modification of the culture method reported by Trowell [29]. MDA-MB-231 human breast cancer cells were cultured in MEM (Lonza) supplemented with 10% (v/v) fetal bovine serum (Gibco), 100 unit/ml penicillin and 100 ug/ml streptomycin. Cells were grown to confluence at 37 °C in a humidified atmosphere containing 5% CO_2_ in air.

### *In situ* hybridization

Cultured specimens were fixed overnight in 4% paraformaldehyde in phosphate buffered saline (PBS). For *in situ* hybridization, the specimens were treated with 20 μg/mL proteinase K for 5 min at room temperature [30]. Antisense RNA probes were labeled with digoxigenin (Roche). MiR-137 locked nucleic acid (LNA) modified probe was synthesized in Exiqon. After *in situ* hybridization, the specimens were cryosectioned at a thickness of 12 μm. At least 20 specimens were examined for each stage.

### Histology and IHC

Samples were fixed in 4% paraformaldehyde in phosphate buffered saline (PBS) and then embedded in paraffin using standard procedures. Serial paraffin sections (4-μm thickness) were prepared, and individual slides were stained with haematoxylin and eosin. Antigen retrieval was achieved by citrate buffer, pH 6.0. After antigen retrieval, immunohistochemical analyses were performed using the DakoCytomation Envision System (using horseradish peroxidase with diaminobenzidine enhancer) (Dako, CA, USA) according to the manufacturer's instructions. The following primary antibodies were used for immunostaining or immunofluorescence: Ki67 (Thermo Scientific, USA), EGFP (Novus, USA), CD31 (BD Pharmingen, USA), vWF (Millipore, USA), VEGF (Santa Cruz Biotechnology, Inc., USA), Runx3 (Abcam, USA), E-cadherin (R&D systems, USA), and Vimentin (Thermo Scientific, USA). Alexa Fluor-conjugated secondary antibodies (Invitrogen, Carlsbad, CA, USA) were used for immunofluorescent staining. The stained sections were examined with a Leica MD5500D and a Zeiss LSM700.

### Quantification of Ki67-positive cells

Ten slides that contained five sections each were used to determine the number of total cells and Ki67-positive cells, and 15 sections were chosen at random from the 10 slides. The number of cells was counted in an area of 100 × 100 μm. After counting the proliferating cells, we corrected the counting results using Abercrombie's method [36].

The equations used is

$$P=A\frac{M}{L+M}$$

P is the average number of nuclear points per section, A the crude count of number of nuclei seen in the section, M the thickness of the section and L is the average length of the nuclei. Data were expressed as the mean ± S.D.

### Evaluation and statistics of IHC staining

Pictures were taken by the confocal microscope (Carl Zeiss LSM700, Germany). The positive pixels of images were counted by the software Leica Application (Leica Microsystems, Germany). All parameters of the image acquisition were kept the same to allow comparison. Data were expressed as mean ± SD. The mean expression levels were compared between the experimental and control groups using ANOVA (SPSS 10.0), with a probability value of *P* < 0.05 (Tukey's HSD test) being considered statistically significant.

### Luciferase assay

For the luciferase assay, the full length 3′UTRs of *Tac1* genes (from 686nt to 1230nt of mRNA) were amplified from E13.5 mouse genomic DNA by PCR using sense and antisense primers carrying an *XhoI* restriction site and a *NotI* restriction site, respectively. The 3′UTR of the *Tac1* gene was then cloned downstream of the Renilla luciferase gene in the psiCHECK^TM^-2 vector (Promega) between the *XhoI* and *NotI* sites. A mutant *Tac1* 3′UTR was synthesized using the QuikChange Lightning Multi Site-Directed Mutagenesis Kit (Agilent Technologies). Primer sequences used for cloning and mutagenesis are reported in Supplementary Figure S6. Cos-7 cells, a fibroblast-like cell line derived from monkey kidney tissue, were cultured in RPMI 1640 Medium (Gibco) supplemented with 10% (v/v) fetal bovine serum (Gibco) and 100X GlutaMax^TM^-I (Gibco), without penicillin and streptomycin antibiotics. After cells grew to 80-90% confluence, wild type (psiCHECK^TM^-2 Tac1) or mutant (Mut) reporter vectors were co-transfected into Cos-7 cells with or without a miR-137 expressing lentiviral vector using FuGENE^®^H (Promega) and salmon sperm DNA (ssDNA, Sigma). A scrambled miRNA was used as control. Renilla and Firefly luciferase activities were measured 24 h and 48 h after transfection. Firefly and Renilla luciferase activities were measured using the dual-luciferase reporter system (Promega) on a GLOMAX^TM^ 20/20 Luminometer (Promega). The data generated were expressed as relative ratios by normalizing Renilla luciferase readings to Firefly luciferase readings. All transfection experiments were performed in triplicate.

### Western blotting analyses

Mammary gland tissue and MDA-MB-231 cells treated with the scrambled miRNA and miR-137-expressing lentiviral vectors underwent lysis by sonication (Nextadvance) in radio-immunoprecipitation assay (RIPA) buffer (50 nM Tris pH 7.5, 150 mM NaCl, 1 mM EDTA, 1% Triton X-100). Anti-p53 (Santa Cruz Biotechnology, Inc., USA) antibody was used. Horseradish peroxidase conjugated secondary antibodies (Santa Cruz Biotechnology, Inc., USA) were used, and the protein bands were visualized by enhanced chemiluminescence (Amersham Biosciences, USA). α-tubulin expression served as an internal control.

## SUPPLEMENTARY MATERIAL FIGURES


