# Supplementary material for: A contrasting function for miR-137 in embryonic mammogenesis and adult breast carcinogenesis
Source: Oncotarget. 2015 May 20;6(26):22048–59. doi: 10.18632/oncotarget.4218 (PMC4673145; doi:10.18632/oncotarget.4218)
Supplement: Supplementary file 1 [file oncotarget-06-22048-s001.pdf]

A contrasting function for miR-137 in embryonic mammogenesis and adult breast carcinogenesis

Supplementary Material

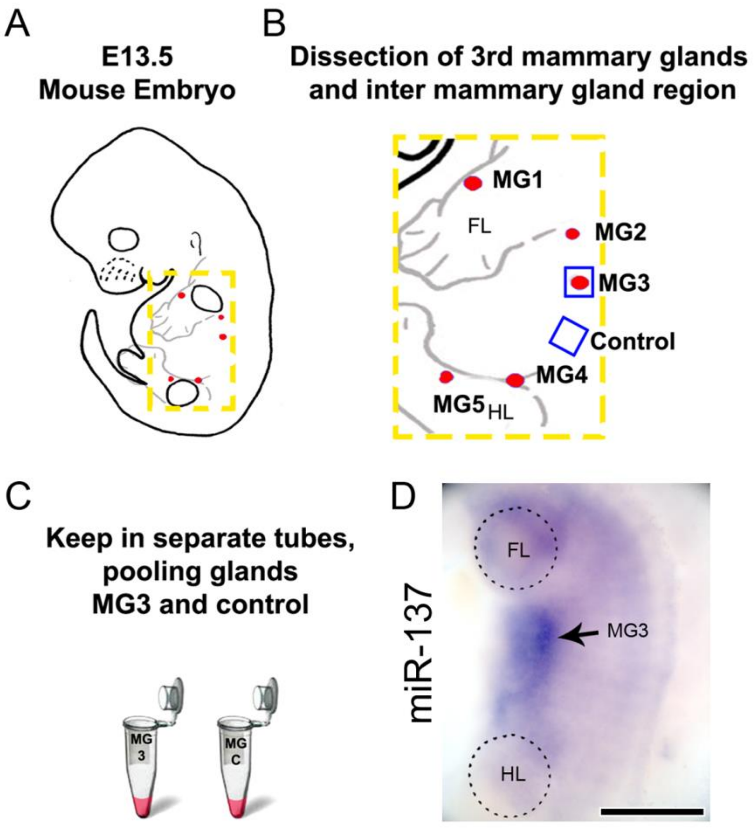

Figure S1: Experimental procedure of isolating 3rd mammary bud and mammary flank tissue.

(A) E13.5 ICR mouse embryos are used for microRNA macroarray and micro-array. (B) Blue boxes indicate the regions isolated to compare 3rd mammary gland and mammary flank. (C) 3rd mammary gland and mammary flank tissues were collected and kept separate for performing the microarray analysis. (D) MiR-137 is highly expressed in the 3rd mammary gland forming region (arrow). FL, fore limb; HL, hind limb; MG, mammary gland; Scale bar, 1 mm.

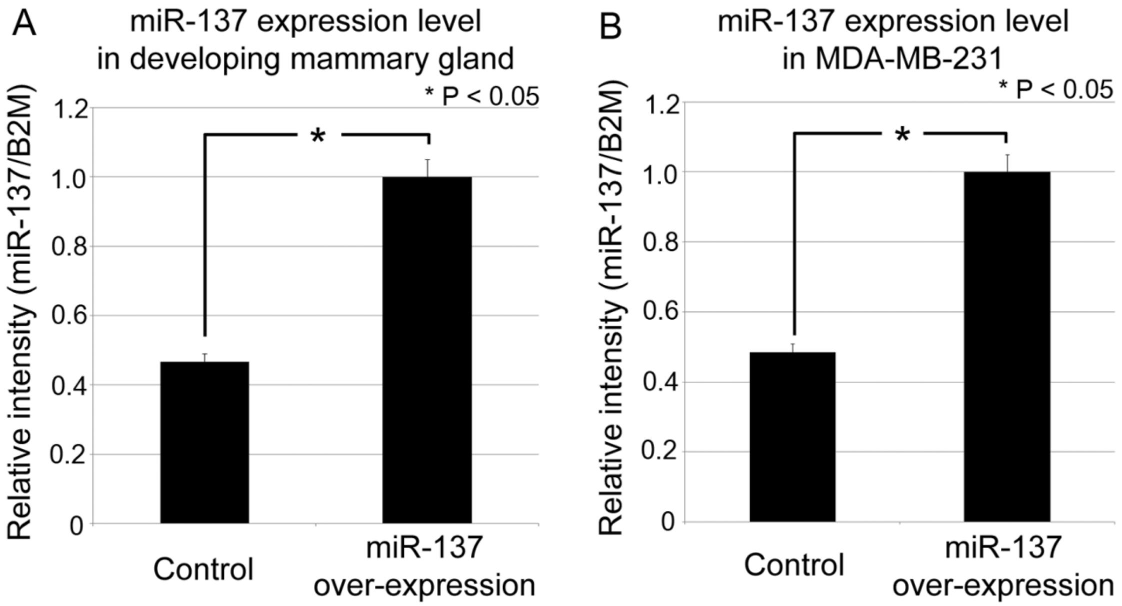

Figure S2: Efficiency of miR-137 over-expression by RT-qPCR.

(A) Level of miR-137 over-expression in developing mammary gland. (B) Level of miR-137 in MDA-MB-231 cell.

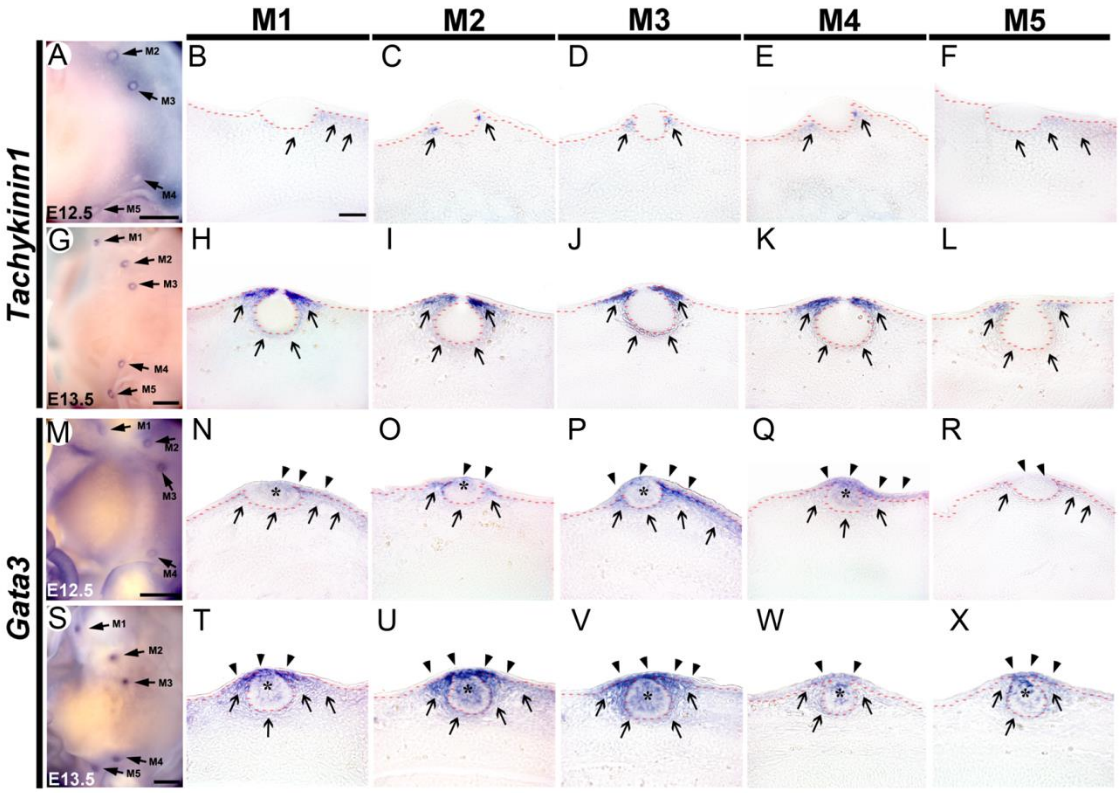

Figure S3: *Tac1* and *Gata3* expression patterns during mouse mammary gland development.

(A-X) In order to confirm expression patterns of *Tac1* and *Gata3*, we performed *in situ* hybridization at E12.5 and E13.5. (A) At E12.5, a ring-like shape of *Tac1* expression pattern is detected in 2nd to 4th mammary bud. (B, F) *Tac1* is also weakly expressed in mesenchyme dorsal to 1st and 5th mammary buds. (C-E) Strong *Tac1* expression detected in mammary mesenchyme at the margin of mammary epithelium and responsible for the ring of expression seen in whole mounts. (G) At E13.5, the ring-like shape of *Tac1* expression observed in all mammary buds 1st to 5th. (H-L) *Tac1* expressed mammary mesenchyme surrounding mammary epithelium. Strong *Tac1* expression detected in mesenchyme beneath nipple forming region of epithelium. (M) *Gata3* expressed in mammary buds at E12.5. (N-R) *Gata3* expressed both in mammary epithelium and mesenchyme at E12.5. (N) In 1st mammary gland, similar levels of *Gata3* expression observed in mammary epithelium and mesenchyme dorsal to mammary bud, weakly

expressed in ventral mesenchyme at E12.5. (O) *Gata3* detected in both 2nd mammary epithelium and surrounding mesenchyme at E12.5. Strong *Gata3* expression observed in mesenchyme beneath nipple forming region of epithelium. (P) In 3rd mammary bud, *Gata3* strongly expressed in dorsal region of mammary epithelium and mesenchyme. (Q) *Gata3* expression more strongly observed in mammary epithelium dorsally compared to mesenchyme at E12.5. (R) In 5th mammary bud, weak *Gata3* expression detected in mammary epithelium and mesenchyme compared to other mammary buds. (S) Stronger *Gata3* expression observed in E13.5 mammary bud compared to E12.5. (T) *Gata3* expressed both in epithelium and mesenchyme of 1st mammary bud near nipple forming region at E13.5. (U-V) Similar *Gata3* expression pattern observed in 2nd to 3rd mammary bud compared with 1st mammary bud. (W) In 4th mammary bud, *Gata3* more weakly expressed both in mammary epithelium and mesenchyme. (X) In 5th mammary bud, *Gata3* expressed in mammary bud, and higher levels in dorsal mesenchyme still present. Black arrows, mesenchymal gene expression; Arrowheads, epithelial gene expression; Asterisks, gene expression in mammary bud, M, mammary bud; Scale bar, B-F, H-L, N-R, T-X, 100μm, A, G, M, S, 1mm.

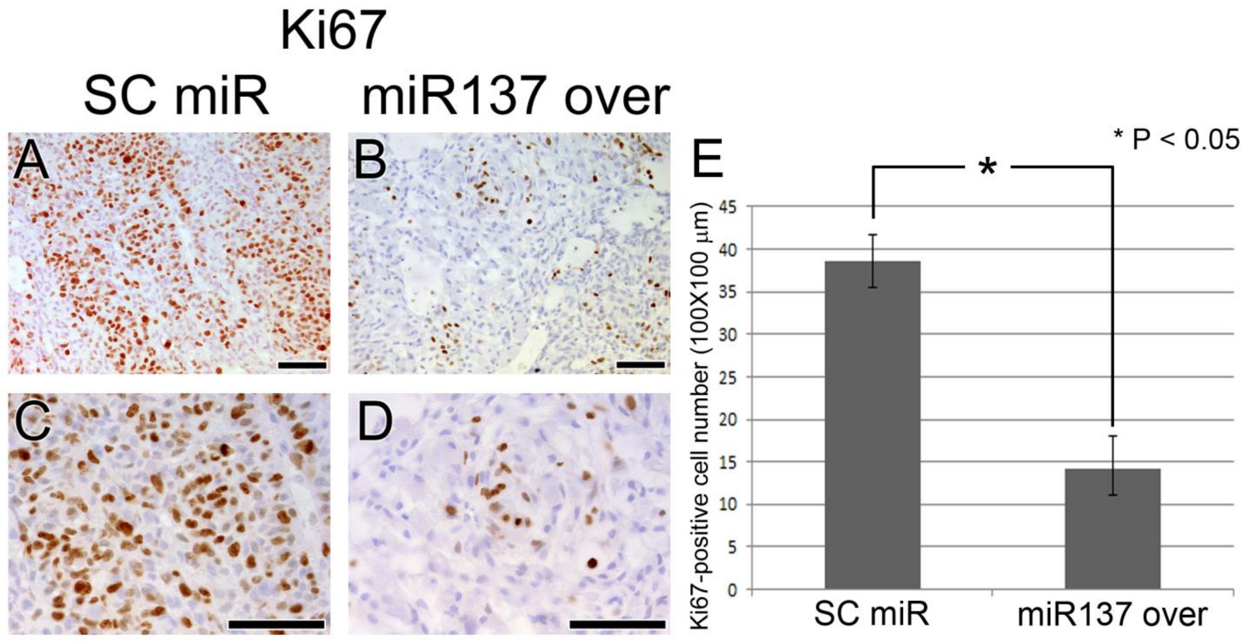

Figure S4: Cell proliferation in tumours formed by MDA-MB-231 cells over-expressing miR-137. (A, C) Large numbers of Ki67 positive proliferating cells are observed with scrambled miRNA treatment. (B, D) Cell proliferation is inhibited by over-expressed miR-137. (E) Ki67-positive cells are reduced in number in miR-137 over-expression group compared to control. Scale bar, 100 μm.

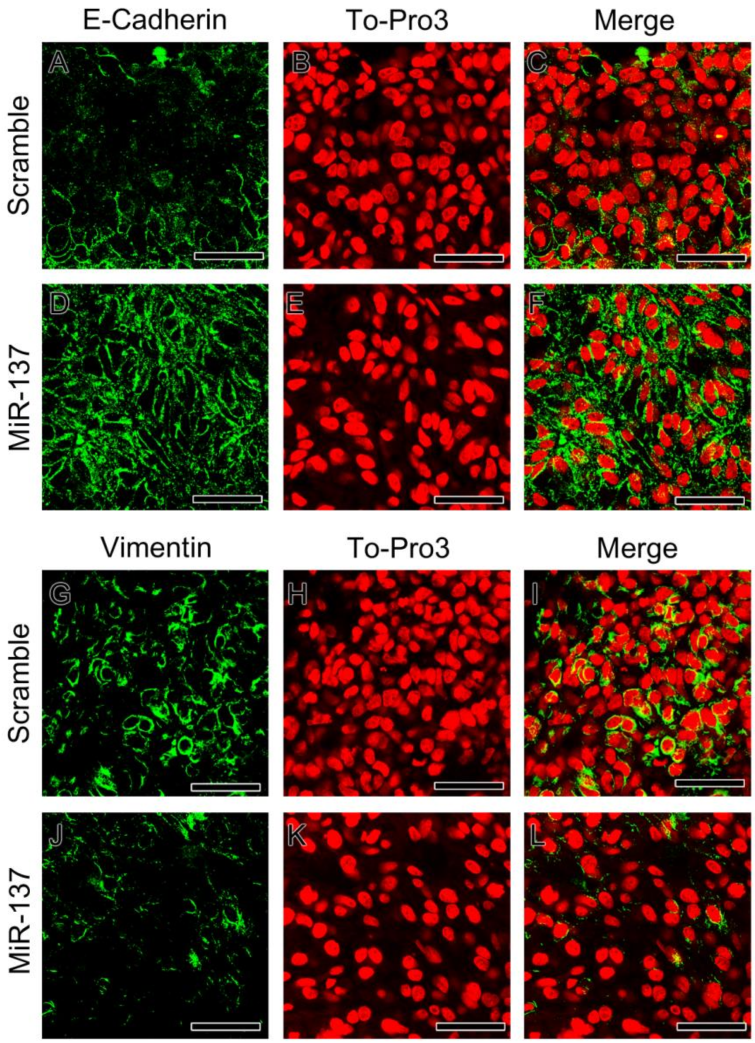

Figure S5: Altered expression of EMT markers in tumours formed by MDA-MB-231 cells over-expressing miR-137.

(A-C) Epithelial marker E-cadherin is expressed at low levels in tumours formed by cells over-expressing scrambled miRNA. (D-F) After miR-137 over-expression, E-cadherin is tightly expressed between cells in the tumour. (G-I) Mesenchymal marker Vimentin is strongly expressed in tumours formed by cells over-expressing scrambled miRNA. (J-L) Vimentin expression is markedly reduced in tumours formed by cells over-expressing miR-137. Scale bar, 100 μm.

Name	Forward primers, 5′ to 3′	Reverse primers, 5′ to 3′
Tac1	CCGCTCGAGACCCCTGAACGCACTATCTATT	GGAACGCGCCGCGGCCGCTCCTGAAGATACATTCTTTTATT
Mut	TGTAATTTCACGCGAGCACAGTGATGAAGG	TCACTGTGCTCGCGTGAAATTACAACTAATTTCTACTTCTGGGG

Figure S6: Primers used for cloning and mutagenesis for luciferase assay.
